# Carotid-Cavernous Sinus Fistula Masquerading as Thyroid Eye Disease

**DOI:** 10.7759/cureus.14261

**Published:** 2021-04-02

**Authors:** Daniel B Azzam, Sanja G Cypen, Jordan R Conger, Jeremiah P Tao

**Affiliations:** 1 Department of Ophthalmology, Division of Oculofacial Plastic & Orbital Surgery, Gavin Herbert Eye Institute, University of California, Irvine, Irvine, USA

**Keywords:** thyroid eye disease, carotid, cavernous sinus, fistula, proptosis, orbital inflammation, orbitopathy

## Abstract

A 29-year-old man with a medical and social history notable for smoking presented with progressive orbital congestion, conjunctival injection, and extraocular muscle enlargement consistent with thyroid eye disease (TED). On ophthalmologic examination, tortuous episcleral vessels and blood in Schlemm’s canal on gonioscopy clued an alternative diagnosis. Cavernous sinus enhancement on computed tomography also suggested a retro-orbital process. Digital subtraction angiography confirmed a low-flow indirect carotid-cavernous fistula (CCF). He subsequently underwent endovascular embolization treatment. Ocular symptoms resolved by seven weeks, and he remained ocular symptom free at six months. Eye redness and proptosis frequently cause patients to seek medical attention. In the absence of a mass or signs of infection, TED is high on the differential, especially with a smoking history and even with normal thyroid parameters. However, CCF may lurk; the authors describe key diagnostic features and management.

## Introduction

First classified by Barrow et al. in 1985, carotid-cavernous fistula (CCF) is a rare cerebrovascular connection between the intracranial carotid arterial system and the cavernous sinus [1]. The diagnosis of CCF may be challenging. Several of the symptoms, including diplopia, progressive proptosis, conjunctival injection, and ophthalmoplegia, mimic thyroid eye disease (TED). While normal thyroid parameters do not rule out TED, subtle ophthalmologic findings can suggest the correct diagnosis of CCF. We present a case of CCF initially misdiagnosed as TED and describe the key clinical findings and management.

## Case presentation

A 29-year-old man presented with a one-year history of right conjunctival injection, progressive proptosis, and diplopia. Past medical history and social history included self-limited Bell’s palsy five years prior and 15-pack-year history of smoking. He denied any major trauma but four years earlier had suffered a contusion of the right periorbital region after a minor motor vehicle crash for which he did not seek medical attention. Review of the systems was negative for vision loss, pain, fever, headache, weight loss, fatigue, or heat intolerance. One year ago, an outside provider diagnosed him with TED, for which he was sent to our institution for management.

**Figure 1:**
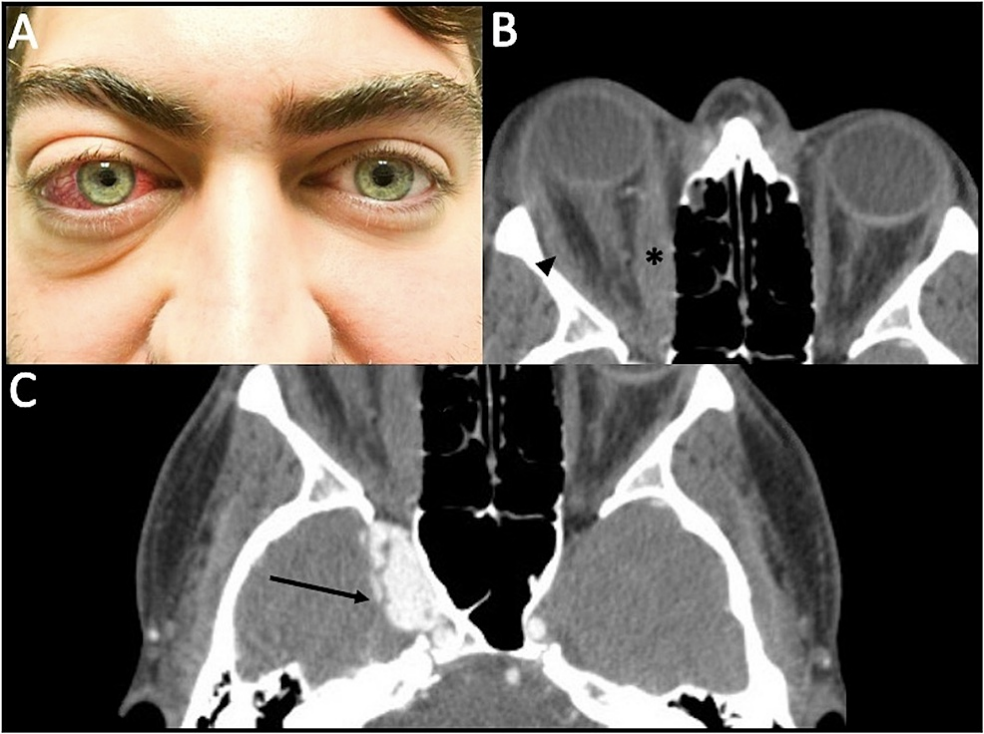
Pre-operative external ocular examination and orbital imaging. (A) External photograph demonstrating proptosis, periorbital edema, and diffuse conjunctival injection of the right eye. (B) Computed tomography scan of the orbits (axial view) demonstrating right-sided proptosis, asymmetric enhancement, as well as enlargement of the right medial (asterisk) and lateral rectus muscles (arrowhead). (C)  Computed tomography scan of the orbits and head showing enhancement of the right cavernous sinus (arrow).

On ophthalmologic examination, visual acuity measured 20/20 in each eye. Intraocular pressures were normal (21 and 19 mmHg in the right and left eyes, respectively). No pupillary abnormality or relative afferent pupillary defect was detected. External examination revealed right proptosis, periorbital edema, and conjunctival injection (Figure 1A). Extraocular movements demonstrated generalized ophthalmoplegia of the right eye most noticeable on abduction. Left eye movements were full.

Computed tomography (CT) scan of the orbits revealed enlarged extraocular muscles that, with the smoking history and clinical findings, corroborated a suspicion for TED (Figure 1B). Serum thyroid studies were normal (thyroid stimulating hormone: 2.37 mIU/L, T3: 125 ng/dL, and T4: 1.0 ng/dL), but euthyroid TED was a leading presumptive diagnosis.

Slit lamp biomicroscopy of the right eye showed dilated and tortuous episcleral vessels. Gonioscopy revealed blood in Schlemm’s canal. Retinal examination demonstrated dilated, tortuous vessels. The left eye examination was unremarkable. Attention to retro-orbital and intracranial aspects of the CT revealed right cavernous sinus enhancement (Figure 1C). These findings prompted suspicion for an intracranial vascular abnormality for which angiography was indicated.

**Figure 2:**
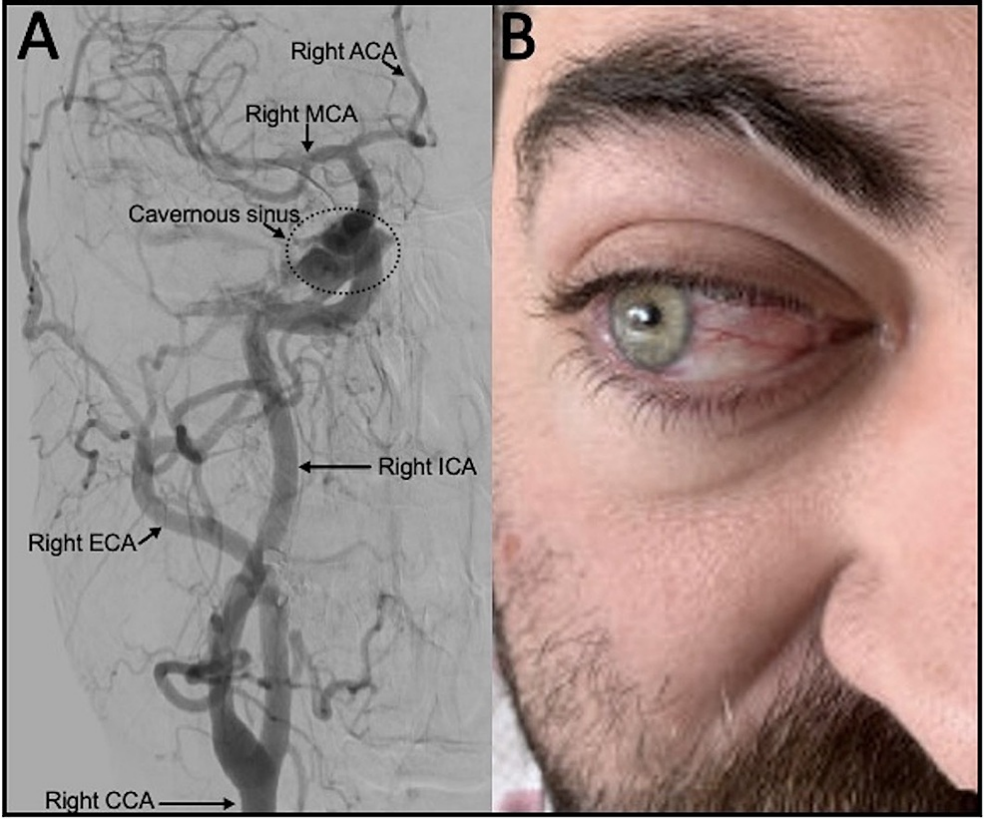
Angiography and post-operative external ocular examination. (A) DSA anterior-posterior view demonstrating right CCA injection before embolization. Opacification of the cavernous sinus (dotted circle) from the right ICA and ECA via branches of the right internal maxillary artery leading to backflow of venous drainage from the cavernous sinus into the right SOV, consistent with an indirect ICA-ECA CCF. (B) Post-operative external photo one day after embolization showing improvement of conjunctival injection and abduction. DSA, digital subtraction angiography; CCA, common carotid artery; ICA, internal carotid artery; ECA, external carotid artery; SOV, superior ophthalmic vein; CCF, carotid-cavernous fistula; ACA, anterior cerebral artery; MCA, middle cerebral artery

Digital subtraction arteriography (DSA) redemonstrated cavernous sinus opacification arising from branches of the right internal carotid artery (ICA) and external carotid artery (ECA). Flow reversal of the cavernous sinus into the right superior ophthalmic vein was consistent with a low-flow ICA-ECA CCF (Figure 2A). The patient subsequently underwent endovascular embolization of the CCF with the liquid embolic system, Onyx® (EV3, Irvine, CA, USA).

Examination one day later revealed improvements in conjunctival injection and abduction (Figure 2B). Three weeks post-operatively, proptosis improved dramatically. At seven weeks, the motility deficit and conjunctival injection resolved. At six months, he continued to have no central nervous system (CNS), orbit, or ocular issues.

## Discussion

CCF is a rare, sight- and CNS-threatening disorder characterized by abnormal communication between the cavernous sinus and the ICA, ECA, or their branches [2]. CCFs may be direct high-flow shunts between the ICA and cavernous sinus and associated with trauma in young males [1,2]. Indirect low-flow shunts are typically spontaneous in older females and occur between the cavernous sinus and branches of the ICA, ECA, or both [1,2]. Rarely, trauma may induce indirect CCF [3].

The clinical presentation of CCF may mimic TED. The differential includes conjunctivitis, scleritis, glaucoma, vascular abnormality, idiopathic orbital inflammation, and orbital malignancy [4]. CCFs may manifest with ocular bruit, exophthalmos, conjunctival injection, and ophthalmoplegia (50-85% abducens nerve) [4]. Vision loss can result from exposure keratopathy, corneal ulceration, retinal artery occlusion, or glaucomatous optic nerve damage from venous congestion. CNS injury or death is possible, although rare from CCF.

Unilateral or bilateral progressive orbital congestion, proptosis, conjunctival injection, extraocular muscle enlargement, and smoking history are also hallmarks of TED, a much more common diagnosis than CCF. Importantly, normal thyroid parameters do not rule out TED as 5-10% of the cases are euthyroid [5].

Tortuous episcleral vessels and blood in Schlemm’s canal, while possible secondary to TED, suggested a more serious cause for orbital venous outflow abnormality. Cavernous sinus enhancement on CT also clued a retro-orbital process, and DSA confirmed the diagnosis of indirect ICA-ECA CCF.

Although subtle, history and examination can suggest CCF. Diagnosis is confirmed with neuroimaging. Carotid DSA remains the gold standard, with endovascular embolization the primary treatment [1,2]. Prognosis after endovascular treatment is excellent, with visual acuity preserved or improved in 94% of the cases [4].

## Conclusions

This case highlights a CCF with many shared features with unilateral TED. Eye redness and proptosis are frequent ocular complaints that in the absence of infection are commonly due to TED, but CNS vascular disease may lurk. CCF often manifests with ocular bruit, proptosis, conjunctival injection, and ophthalmoplegia; however indirect CCF may have a more subtle presentation that can mirror inflammatory processes. While normal thyroid parameters do not rule out TED, detailed ophthalmologic examination and neuroimaging are keys to diagnostic accuracy. Timely endovascular embolization treatment of CCF yields an excellent prognosis.
